# Supplementary material for: Development and Validity Evaluation of the Index of Social Work Process in Promoting Social Participation of Welfare Recipients (SWP-PSP) in Japan
Source: Int J Environ Res Public Health. 2025 Sep 20;22(9):1458. doi: 10.3390/ijerph22091458 (PMC12470206; doi:10.3390/ijerph22091458)
Supplement: Supplementary file 1 [file ijerph-22-01458-s001.zip › ijerph-3806705-supplementary.pdf]

Supplementary Materials

Table S1. Concept integration.

Proposed Domain	Extracted from existing Social Work standards	Extracted from Leadership theories
Effective Relationship	1) interpersonal relationship building [16,18]	a) trust building [21-24]<br>b) active listening [22,26]
Deliberative Support	2) support for appropriate decision-making [16,17]	c) suggesting goal options [20,25,26,28]
Positive Feedback	6) building up the confidence of service users including specifying their strengths [16-18]	d) helping gain confidence [25,26,28]
Tailored Information	3) flexibility for changes [16,18]<br>5) tailored service or information [16,17]	e) timely information provision [25, 27,28]
Network Development	4) development and coordination of social resources [16-19]	–

Table S2. Drafts of domains and selected items.

Domain	Items
Effective relationship	•I listen to the client while using verbal (e.g., repeating, questioning) and non-verbal (e.g., nodding) communication skills.<br>•I express that I am caring about the client when they talk about their worries or something unpleasant.<br>•I do not reject the thoughts of the client without considering their values.
Deliberative support	•I logically explain to the client why they have to work based on institutional requirements.<br>•I ask the client what occupations they are interested in when they are not proactive toward work.<br>•I suggest new options (e.g., transitional employment programs) to the client depending on their needs.
Positive feedback	• I verbalize the client's strengths and feedback clearly.<br>• I show compassion to the client by telling them that I am on their side.<br>• I commend the client when they make progress even if it is small.
Tailored information	• I confirm with the client whether they understand what they are expected to do in the procedure.<br>•I confirm with the client whether they can perform the procedures expected of them.<br>•I read the client's expressions regarding any unspoken apprehension.

Network development	•I obtain information or advice from my coworkers when I cannot find social resources which the client can use.<br>•I obtain information or advice from other organizations when I cannot find social resources which the client can use.<br>•I regularly share information with the client's social resources.

We used the term "client" to refer to recipients to generalize the items to other social services.

The process of item revision in the focus group interview

The experts discussed the items in order from domains 1 to 5. They considered that the items in domain 1 were elementary but important communication skills. Some revisions were suggested, e.g., the item "I express that I am caring about the client when they talk about their worries or something unpleasant" was revised to "I listen to the client with an appropriate expression given what they are saying". In addition, they considered that the item "I do not reject the thoughts of the client without considering their values" should be the reverse item. Experts discussed whether the SWP-PSP should include the enforcement power of PLS social workers using the law with regard to the item "I logically explain to the client why they have to work based on institutional requirements". They rejected this idea because they thought the PLS social workers should motivate recipients using techniques based on behavioral science. They recommended that the meaningfulness of work should be explained based on the holistic concept of self-reliance, which consists of three aspects: individuals managing their own health and daily life, participating in society, and engaging in paid work. They also voiced concerns about the item "I suggest new options (e.g., transitional employment programs) to the client depending on their needs". They asserted that the transitional employment programs or voluntary activities are not separate to the employment support which the SWP-PSP aims for because starting with such programs or activities is helpful for achieving holistic self-reliance. They advised that we employed the term "works for diverse social participation". The experts generally agreed with items in domains 3 to 5. Overall, they recommended integrating similar items and employing easier, clear terms.
